
JOURNAL INFORMATION
==============================
NLM Title Abbreviation: Hist Cienc Saude Manguinhos
Journal ID: Hist Cienc Saude Manguinhos
NLM Journal ID: 9513999
Journal ID: hcsm

História, Ciências, Saúde - Manguinhos

ISSN: 0104-5970
EISSN: 1678-4758
Publisher: Casa de Oswaldo Cruz - Fiocruz

ARTICLE INFORMATION
==============================
PMCID: PMC10481632
DOI: 10.1590/S0104-59702023000100034
Article ID: 01500
Article version: 1
Subjects: Reseñas

Covid-19 and neoliberalism

Covid-19 y neoliberalismo

http://orcid.org/0000-0003-3710-2804 Murillo Juan Pablo i
i Facultad de Medicina de San Fernando/Universidad Nacional Mayor de San Marcos. Lima – Peru jpmurillop@gmail.com
Electronic publication date: 2023 Aug 14
Publication date: 2023
Volume: 30
Issue: Suppl 1
Electronic Location ID: e2023034
Product: CUETO Marcos  
Salud en emergencia: historia de las batallas contra las epidemias y la covid-19.
Lima: Taurus. 2022. 264.
License: This is an Open Access article distributed under the terms of the Creative Commons Attribution License, which permits unrestricted use, distribution, and reproduction in any medium, provided the original work is properly cited.
License URL: https://creativecommons.org/licenses/by/4.0/

Figure count: 0
Table count: 0
Equation count: 0
Reference count: 3

==============================

El último libro de Marcos Cueto (2022), *Salud en emergencia*, es probablemente una de las obras más ambiciosas que se han publicado últimamente en el campo de la historia de la salud. Por un lado, intenta desde una mirada global describir los aspectos más importantes de la pandemia de covid-19 y para ello analiza el contexto de la evolución de la salud internacional y sus más importantes instituciones. Reflexiona de forma concurrente, acerca de la forma de construir un relato en el campo de la historia de la salud, tanto a partir de sus vivencias al tomar contacto con su objeto de estudio como a partir de un diálogo con referentes importantes como los historiadores de la medicina Roy Porter y Elizabeth Fee. Un elemento a destacar, es el hecho de ser un ensayo escrito desde un territorio personal, desde la mirada de un "peruano nómade" – como se llama uno de los apartados del libro – que transita y reflexiona en clave global sobre las epidemias, con un pie anclado en el Perú, en sus raíces, en sus innumerables frustraciones como país, alimentado también por el enorme dolor y decepción que nos produjo como sociedad la gran tragedia que significó la pandemia de covid-19.

El centro del libro, es una rigurosa crítica al neoliberalismo y como este, a través de las pandemias, expresa sus características más perversas, un poco en la línea que iniciaron Ted Schrecker y Clare Bambra (2015) cuando desarrollan sus reflexiones sobre las "epidemias del neoliberalismo". De una manera muy didáctica Marcos Cueto toma la pandemia de covid-19 como un estudio de caso y desde diferentes aproximaciones nos muestra cómo esta emergencia global, de una manera trágica, nos regresa a la vieja discusión de la medicina social del siglo XIX y de la primera mitad del siglo XX, de cómo la exclusión y la desigualdad social forman parte del núcleo causal de la aparición y propagación de las enfermedades. Además, analiza cómo en momentos de crisis, en un momento de la historia de la humanidad donde se ha llegado a los mayores niveles de exclusión y desigualdad, la bio-medicina y las técnicas de gestión sanitaria derivadas de ellas, han sido incapaces de contener y gestionar la emergencia pandémica (Murillo, 2022). Para ello, en un lenguaje sencillo el autor recurre a una serie de “viñetas” donde, a partir de algunos aspectos puntuales observados en distintas partes del mundo, describe de manera didáctica las consecuencias del neoliberalismo en diversas sociedades y los distintos mecanismos que configuran la vivencia de la pandemia en dichos países. Es importante señalar que el autor no solamente describe los procesos de deterioro de los sistemas de salud por las políticas neoliberales y la enorme crisis de liderazgo a nivel político observada en la pandemia, sino que contrasta con momentos en los cuales fuimos capaces de desplegar políticas alternativas en el campo de la salud pública, a partir de las políticas que desarrolló el Estado brasileño en torno a la provisión universal de tratamientos antirretrovirales para el VIH.

Un indudable aporte de esta obra es sin duda su orientación a constituir un nuevo universo de lectores, ciudadanos informados en conocer la complejidad de las diversas articulaciones globales y la diversidad de respuestas locales frente a la pandemia y explorar la posibilidad de construir mejores sociedades, más inclusivas y solidarias. Sin duda los profesionales y especialistas en la salud tienen en esta obra una valiosa fuente de información y una visión muy personal de cómo podemos construir un relato histórico que nos permita entender los desafíos que implica la mejora del estado de salud de nuestras poblaciones.

Un elemento que encandila el relato, es la relación personal que el autor tiene con su objeto de estudio. Muchas veces escrito en primera persona, este refiere su percepción y contraste personal entre los discursos y su experiencia subjetiva, a veces incluso cercana al relato etnográfico, Marcos Cueto nos muestra su interacción con sus fuentes, la serena evaluación de los discursos y su mirada lúcida de los diferentes contextos que rodean al hecho que quiere describir y comprender. Finalmente nos lleva a los límites, que a la luz de la pandemia, se observan el concepto de salud global y señalándonos distintos caminos alternativos, entre ellos el nuevo paradigma de salud planetaria.

En resumen, un libro que nos brinda interesantes claves para comprender en estos tiempos grises la grave crisis sanitaria, social y política por la que pasan nuestros países de América Latina. Una contribución a la apuesta que a pesar de la magnitud de los desafíos siempre hay espacios para avanzar, a la construcción de mundos alternativos y a un mejor futuro para las siguientes generaciones si recogemos las enormes enseñanzas que nos deja esta pandemia.


REFERENCIAS

CUETO Marcos Salud en emergencia: historia de las batallas contra las epidemias y la covid-19 Lima Taurus 2022
MURILLO Juan Pablo Sindemia o violencia estructural: el regreso a una vieja discusión sobre la salud y la enfermedad Anales de la Facultad de Medicina 83 2 83 86 2022
SCHRECKER Ted BAMBRA Clare How politics makes us sick: neoliberal epidemics New York Palgrave Macmillan 2015
